# Supplementary material for: Monte Carlo profile confidence intervals for dynamic systems
Source: J R Soc Interface. 2017 Jul 5;14(132):20170126. doi: 10.1098/rsif.2017.0126 (PMC5550967; doi:10.1098/rsif.2017.0126)
Supplement: Supplementary Text [file rsif20170126supp1.pdf]

Supplementary text for "Monte Carlo profile confidence intervals for dynamic systems"

E. L. Ionides[1], C. Breto[1], J. Park[1], R. A. Smith[2] and A. A. King[3,4]

Departments of Statistics[1], Bioinformatics[2], Ecology & Evolutionary Biology[3] and Mathematics[4]
The University of Michigan, Ann Arbor, Michigan, USA.
email: ionides@umich.edu

Published by *Journal of the Royal Society Interface.*

Supplementary Content

S1 Inference for nonlinear partially observed spatiotemporal systems **S-2**

S2 Comparing MCAP with Fisher information and a bootstrap **S-3**

S3 Implementation of the MCAP algorithm in R **S-4**

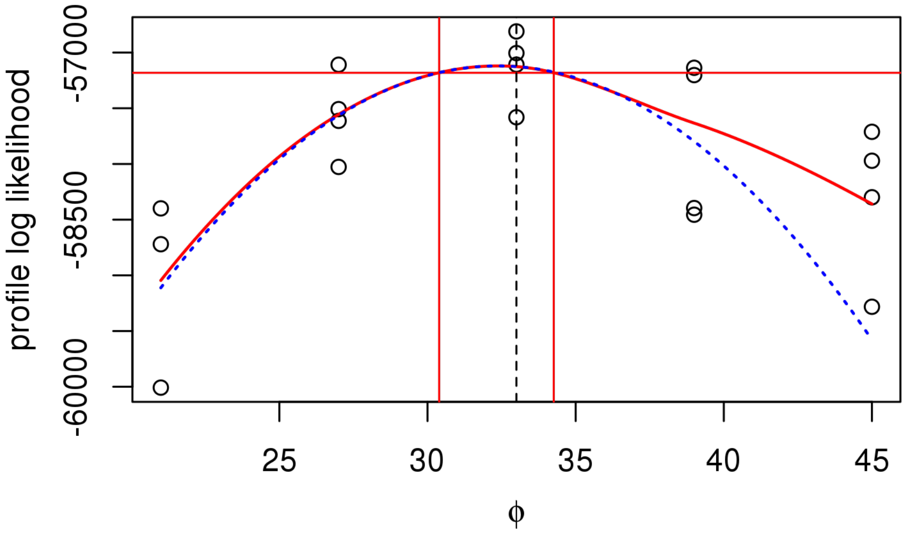

Figure S-1: Profile likelihood for a spatiotemporal measles transmission model with twenty metapopulations. The profile parameter describes the contact rate within each metapopulation. For this figure, the data were simulated from a fitted model, and so the true parameter can be shown (dashed black vertical line). The smoothed profile likelihood and corresponding MCAP 95% confidence interval are shown as solid red lines. The quadratic approximation in a neighborhood of the maximum is shown as a dotted blue line.

S1 Inference for nonlinear partially observed spatiotemporal systems

We consider statistical inference for a mechanistic spatiotemporal model, meaning a collection of nonlinear partially observed spatially coupled Markov process. Appropriately designed sequential Monte Carlo (SMC) schemes can successfully carry out Monte Carlo likelihood evaluation for general partially observed spatiotemporal processes of modest dimension. Park and Ionides (2017) developed such an SMC scheme and then employed iterated filtering methodology (Ionides et al., 2015) to maximize the likelihood. Here, we are not immediately concerned with the details of the model and the SMC algorithm but rather with indicating how MCAP methodology may play a role in this computationally demanding inference problem by enabling statistical conclusions to be drawn from a noisy Monte Carlo profile.

Figure S-1 shows an estimated likelihood profile for a parameter ϕ corresponding to the contact rate between individuals (denoted as β by Park and Ionides, 2017) when fitting a ten parameter model to pre-vaccination measles incidence in 20 cities in the United Kingdom. This profile corresponds to a simulation test of the methodology of Park and Ionides (2017) in which the true parameter is known. For this computation, only five distinct parameter values were used when computing the profile. The default smoothing parameter $\lambda = 0.75$ was too small in this case, since the local quadratic fit by the smoother at the maximum placed almost all its weights on only three distinct parameter values. The resulting numerical instability was avoided by taking $\lambda = 1$. For this analysis, the profile cutoff adjusted for Monte Carlo uncertainty is $\delta = 61.6$, and we see that

the Monte Carlo variability $\text{SE}_{\text{mc}} = 1.00$ in the parameter estimate greatly exceeds the statistical variability $\text{SE}_{\text{stat}} = 0.18$. Evidently, the simulated spatiotemporal data have a considerable amount of information about the parameter ϕ, but extracting that information in a statistically efficient way is complicated by the computational challenge of working with the likelihood of a nonlinear partially observed spatiotemporal process.

S2 Comparing MCAP with Fisher information and a bootstrap

Two alternative approaches to generating confidence intervals based on a maximum likelihood estimator are observed Fisher information and the bootstrap method. We discuss each of these in turn.

Observed Fisher information requires computation of a second derivative which is not directly available in the context of plug-and-play methodology. Numerical estimation of this derivative is equivalent to estimation of a quadratic approximation to the log likelihood at the maximum. Further, rather than estimating all $p(p+1)/2$ terms in the full observed Fisher information, it is sufficient for the construction of marginal confidence intervals to estimate the p terms of the marginal observed Fisher information for each parameter. In this context, implementation of observed Fisher information becomes equivalent to using the cutoff δ of Section 3 on the local quadratic approximation $\breve{\ell}^Q(\phi\,;\,y^*)$. We therefore refer to the Monte Carlo observed Fisher information confidence interval estimator as the quadratic estimator. We expect that, so far as the log likelihood deviates from being quadratic, the profile estimator MCAP may have a statistical advantage over the quadratic estimator. Table S-1 shows that, on our toy example, the quadratic estimator leads to less precise (wider) confidence intervals with similar coverage to the MCAP estimator.

Bootstrap confidence intervals can be computed via the following steps.

1. Evaluate the Monte Carlo estimator, $\breve{\theta}(y^*)$.
2. Simulate B datasets $y_{1:B}$ from the model with $\theta = \breve{\theta}(y^*)$.
3. Evaluate the Monte Carlo estimator on y_b to obtain $\breve{\theta}_b = \breve{\theta}(y_b)$ for $b \in 1\,{:}\,B$.
4. For each component of the vector θ, use the corresponding $\alpha/2$ and $1-\alpha/2$ quantiles of $\breve{\theta}_{1:B}$ as an approximate confidence interval.

The bootstrap adjusts appropriately for Monte Carlo error in the estimator, since the Monte Carlo error is simply included as part of the total error in re-evaluating the estimator on simulated data. From the point of view of asymptotic statistical efficiency, observed Fisher information (and

	Exact profile	MCAP profile	Bootstrap	Quadratic
Coverage %	94.3	93.4	93.3	93.3
Mean width	0.78	0.88	0.94	0.92

Table S-1: Comparison of methodologies to construct an approximate 95% confidence interval for the toy example of Section 5. The exact profile relies on an exact likelihood estimator. The other three methods build on the same Monte Carlo estimator and optimizer. Results are based on 10^4 replications, each involving 30 Monte Carlo maximizations for simulated datasets of length 50.

therefore its close cousin, profile likelihood estimation) have higher order accuracy properties not shared by the bootstrap method (Lindsay and Li, 1997). However, in our context, computational efficiency is at least as important. We suppose that computational effort is dominated by the cost of evaluating the Monte Carlo estimator. The bootstrap method spends $B/(B+1)$ of its computational effort maximizing likelihoods for simulated data, rather than the actual data, and therefore gives less opportunity for the Monte Carlo exploration to discover difficult-to-find features of the likelihood surface for the actual data. Further, each of the B bootstrap replications cannot help to reduce the Monte Carlo error in the original point estimate so, unlike for profile methodology, the Monte Carlo error does not vanish as B becomes large. Table S-1 reports a bootstrap confidence interval for ϕ using the same computational effort as used for the MCAP and quadratic approximation methods, i.e., setting $B = K - 1 = 29$. We see that the bootstrap method is inferior to MCAP on our toy example. However, this particular example does not give the bootstrap method opportunity to benefit from its computational advantage of constructing confidence intervals for all components of θ simultaneously. If one is equally interested in all p parameters, the bootstrap method can employ $B = pK - 1$ to compare with the K maximizations used for each profile. Alternatively, the bootstrap method can allocate additional Monte Carlo effort on each maximization. Thus, the comparison in Table S-1 is only appropriate when there are relatively few parameters of primary scientific interest.

S3 Implementation of the MCAP algorithm in R

The following R code carries out the MCAP algorithm described in the main text.

```r
mcap <- function(lp,parameter,confidence=0.95,lambda=0.75,Ngrid=1000){
  smooth_fit <- loess(lp ~ parameter,span=lambda)
  parameter_grid <- seq(min(parameter), max(parameter), length.out = Ngrid)
  smoothed_loglik <- predict(smooth_fit,newdata=parameter_grid)
  smooth_arg_max <- parameter_grid[which.max(smoothed_loglik)]
  dist <- abs(parameter-smooth_arg_max)
  included <- dist < sort(dist)[trunc(lambda*length(dist))]
  maxdist <- max(dist[included])
  weight <- rep(0,length(parameter))
  weight[included] <- (1-(dist[included]/maxdist)^3)^3
  quadratic_fit <- lm(lp ~ a + b, weight=weight,
    data = data.frame(lp=lp,b=parameter,a=-parameter^2)
  )
  b <- unname(coef(quadratic_fit)["b"] )
  a <- unname(coef(quadratic_fit)["a"] )
  m <- vcov(quadratic_fit)
  var_b <- m["b","b"]
  var_a <- m["a","a"]
  cov_ab <- m["a","b"]
  se_mc_squared <- (1 / (4 * a^2)) * (var_b - (2 * b/a) * cov_ab + (b^2 / a^2) * var_a)
  se_stat_squared <- 1/(2*a)
  se_total_squared <- se_mc_squared + se_stat_squared
  delta <- qchisq(confidence,df=1) * ( a * se_mc_squared + 0.5)
  loglik_diff <- max(smoothed_loglik) - smoothed_loglik
  ci <- range(parameter_grid[loglik_diff < delta])
```

```
  list(lp=lp,parameter=parameter,confidence=confidence,
    quadratic_fit=quadratic_fit, quadratic_max=b/(2*a),
    smooth_fit=smooth_fit,
    fit=data.frame(
      parameter=parameter_grid,
      smoothed=smoothed_loglik,
      quadratic=predict(quadratic_fit, list(b = parameter_grid, a = -parameter_grid^2))
    ),
    mle=smooth_arg_max, ci=ci, delta=delta,
    se_stat=sqrt(se_stat_squared), se_mc=sqrt(se_mc_squared), se=sqrt(se_total_squared)
  )
}
```

Supplementary References

Ionides, E. L., Nguyen, D., Atchadé, Y., Stoev, S., and King, A. A. (2015). Inference for dynamic and latent variable models via iterated, perturbed Bayes maps. *Proceedings of the National Academy of Sciences of the USA*, 112:719–724.

Lindsay, B. G. and Li, B. (1997). On second-order optimality of the observed Fisher information. *Annals of Statistics*, 25:2172–2199.

Park, J. and Ionides, E. L. (2017). Particle filter for continuous time partially observed Markov processes in high dimension via intermediate resampling. *In preparation.*
